# Single‐cell RNA‐seq reveals early heterogeneity during aging in yeast

**DOI:** 10.1111/acel.13712

**Published:** 2022-10-01

**Authors:** Jincheng Wang, Yuchen Sang, Shengxian Jin, Xuezheng Wang, Gajendra Kumar Azad, Mark A. McCormick, Brian K. Kennedy, Qing Li, Jianbin Wang, Xiannian Zhang, Yi Zhang, Yanyi Huang

**Affiliations:** ^1^ Biomedical Pioneering Innovation Center (BIOPIC), Peking‐Tsinghua Center for Life Sciences, Beijing Advanced Innovation Center for Genomics (ICG), School of Life Sciences Peking University Beijing China; ^2^ State Key Laboratory of Protein and Plant Gene Research, School of Life Sciences and Peking‐Tsinghua Center for Life Sciences Peking University Beijing China; ^3^ Academy for Advanced Interdisciplinary Studies Peking University Beijing China; ^4^ Departments of Biochemistry, Yong Loo Lin School of Medicine National University of Singapore Singapore Singapore; ^5^ Department of Zoology Patna University Patna India; ^6^ Department of Biochemistry and Molecular Biology, School of Medicine University of New Mexico Health Sciences Center Albuquerque New Mexico USA; ^7^ Autophagy Inflammation and Metabolism Center of Biomedical Research Excellence Albuquerque New Mexico USA; ^8^ Healthy Longevity Programme, Yong Loo Lin School of Medicine National University of Singapore Singapore Singapore; ^9^ Centre for Healthy Longevity National University Health System Singapore Singapore; ^10^ School of Life Sciences, Beijing Advanced Innovation Center for Structural Biology Tsinghua University Beijing China; ^11^ School of Basic Medical Sciences, Beijing Advanced Innovation Center for Human Brain Protection Capital Medical University Beijing China; ^12^ Analytical Chemistry, College of Chemistry Peking University Beijing China; ^13^ Institute for Cell Analysis Shenzhen Bay Laboratory Shenzhen China

**Keywords:** early heterogeneity, iron transport, mitochondrial dysfunction, single cell RNA sequencing, yeast aging

## Abstract

The budding yeast *Saccharomyces cerevisiae* (*S*. *cerevisiae*) has relatively short lifespan and is genetically tractable, making it a widely used model organism in aging research. Here, we carried out a systematic and quantitative investigation of yeast aging with single‐cell resolution through transcriptomic sequencing. We optimized a single‐cell RNA sequencing (scRNA‐seq) protocol to quantitatively study the whole transcriptome profiles of single yeast cells at different ages, finding increased cell‐to‐cell transcriptional variability during aging. The single‐cell transcriptome analysis also highlighted key biological processes or cellular components, including oxidation–reduction process, oxidative stress response (OSR), translation, ribosome biogenesis and mitochondrion that underlie aging in yeast. We uncovered a molecular marker of *FIT3*, indicating the early heterogeneity during aging in yeast. We also analyzed the regulation of transcription factors and further characterized the distinctive temporal regulation of the OSR by *YAP1* and proteasome activity by *RPN4* during aging in yeast. Overall, our data profoundly reveal early heterogeneity during aging in yeast and shed light on the aging dynamics at the single cell level.

## Graphical abstract

Here, we optimize a single‐cell RNA sequencing (scRNA‐seq) protocol to quantitatively study the whole transcriptome profiles of single yeast cells at different ages. Globally there is an increased cell‐to‐cell transcriptional variability during aging. And we also identify a molecular marker of FIT3 (Facilitator of iron transport) that indicates the early aging heterogeneity: FIT3 is highly expressed in the slow‐dividing early age subgroup. In addition, FIT3 deletion expands the replicative lifespan in yeast.

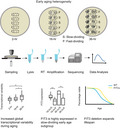

**Abbreviations**
BP: biological processes
CV: coefficients of variation
CV2: squared coefficient of variation
ERCC: external RNA control consortium
FIT3: facilitator of iron transport
GO: Gene Ontology
HVGs: highly variable genes
log2FC: log2FoldChange
OSR: oxidative stress response
PCA: principal component analysis
RLS: replicative life span
scRNA‐seq: single‐cell RNA sequencing
TF: transcription factor
TPM: Transcripts Per Kilobase of exon model per Million mapped reads
UPR: unfolded protein response
WT: wild type

## 1 INTRODUCTION

It has been known for a long time that budding yeast *S. cerevisiae* has limited division potential, only producing a finite number of daughter cells before death (Mortimer & Johnston, 1959). This phenomenon is defined as replicative aging, and the number of daughter cells produced before death is defined as the replicative lifespan (RLS) (Kaeberlein et al., 2007). Owing to its relatively short lifespan, detailed knowledge of its biology and its easy genetic manipulation, *S. cerevisiae* is regarded as an ideal model organism to study aging (Denoth Lippuner et al., 2014). Indeed, many aging genes and signaling pathways initially found in yeast have also been later found to be conserved in other organisms, such as *C. elegans*, *M. musculus*, and even humans (McCormick et al., 2015).

A dilemma of replicative aging research in yeast exists between the rarity of old cells among an exponentially growing population either on a solid agar plate or in liquid media and the large number of pure old cells conventionally required for biochemical, genomic, or transcriptomic analysis. To address this problem, several approaches have been developed to enrich old yeast cells, including magnetic sorting, elutriation, genetic programming, and even computation (Hendrickson et al., 2018; Hu et al., 2014; Leupold et al., 2019; Lindstrom & Gottschling, 2009; Smeal et al., 1996). However, these methods have yet to be successful at simultaneously ensuring both the quantity and purity of the isolated old yeast cells much less distinguishing old but living cells from dead ones. In addition, conventional bulk population analysis of aging yeast cells may likely obscure some specific features within sub‐populations due to the average effect (Zhang et al., 2018). Therefore, a systematic and quantitative investigation of yeast aging at the single‐cell and transcriptome level would be highly valuable.

Here, we developed a single‐cell RNA‐seq approach to study the replicative aging of yeast and quantitatively assessed the heterogeneity between single yeast cells. Instead of partially purifying millions of old cells, exploiting single‐cell technologies enabled us to obtain novel insights into yeast aging from hundreds of single cells with precise ages. By profiling the transcriptomic landscapes of single yeast cells, we observed an increased cell‐to‐cell transcriptional variability and identified key functional biological processes or cellular components that were highly enriched during aging. We also found early heterogeneity during aging indicated by some iron transporter genes, and successfully characterized the distinctive temporal regulation of transcription between slow‐dividing and fast‐dividing age subgroups.

## 2 RESULTS

### 2.1 Isolation of single yeast cells during aging for scRNA‐seq

Single yeast cells from isogenic populations ultimately have different lifespans. In fact, this is a universal phenomenon of aging across species, albeit in different forms and ranges. And previous single‐cell imaging data of replicative aging in yeast have provided evidence of such heterogeneity. For example, when re‐analyzing the single cell imaging data from a previous microfluidic‐based yeast aging study (Video S1, Zhang et al., 2012), we can observe that as early as 8 h after birth, the distribution of generations of single yeast cells had already become dispersed, and the ranges of the distribution gradually increased at 12 and 16 h after birth (Figure S1a), showing that some cells always divided more rapidly than others ever since early in life. These early‐stage cell division dynamics in yeast seems closely associated with replicative age, with a positive correlation between the generations at early time points (8, 12, 16 h) after birth and the RLS at the single‐cell level (*R* = 0.46, 0.64, 0.73; *p* = 9.6 × 10^−5^, 7.7 × 10^−9^, 7.7 × 10^−9^; Figure S1b). This new finding is consistent with the previous report that the division time of single yeast cells early in life is negatively correlated with RLS, and the division time increases dramatically when approaching the end of life (Zhang et al., 2012). It was also reported previously that early in life, the gene expression level of *HSP104*, which encodes a molecular chaperone that maintains proteostasis in yeast, negatively correlates with RLS (Xie et al., 2012; Zhang et al., 2012). Accordingly, after re‐analyzing the single cell imaging data (Zhang et al., 2012), we observed a negative correlation between the generations at early time points during aging and the *HSP104* gene expression level indicated by a GFP tag fused to this gene in single yeast cells (*R* = −0.43, −0.51, −0.56; *p* = 2.8 × 10^−4^, 8.4 × 10^−6^, 7.8 × 10^−7^; Figure S1c). Collectively, these single‐cell imaging data indicate an early heterogeneity of cell divisions during aging in yeast, and that the division dynamics early in life can predict lifespan.

**FIGURE 1:**
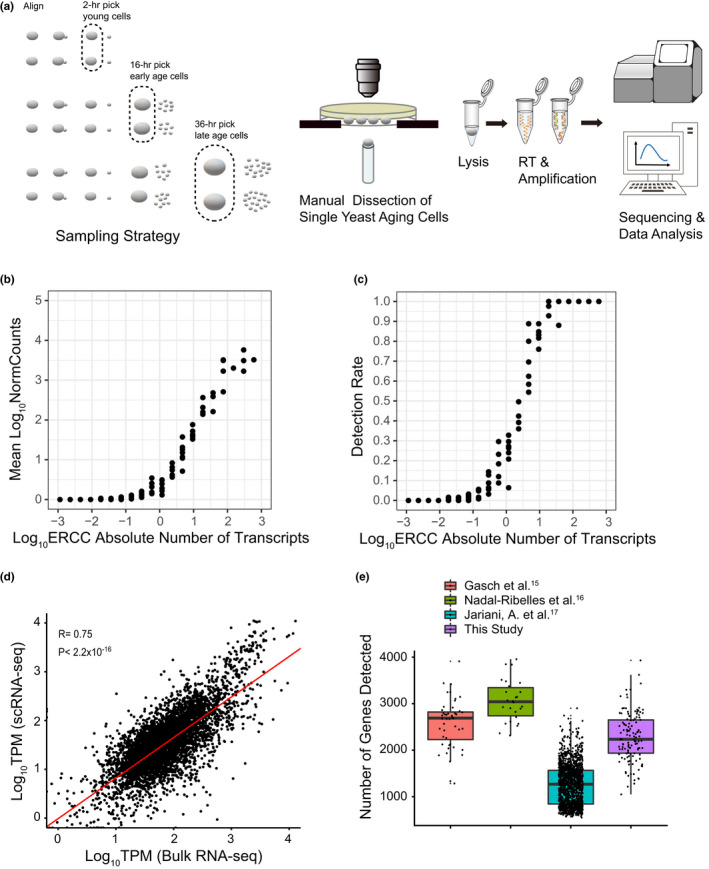
Isolation of single yeast cells during aging for scRNA‐seq. (a) Schematic of the workflow of scRNA‐seq during aging in yeast. Each single yeast aging cell (indicated as gray ellipse in the dashed area) was manually isolated at 2‐, 16‐ or 36‐h after birth, and then placed individually into a single tube prefilled with lysis buffer, followed by modified and optimized Smart‐seq2 (Picelli et al., 2014). (b, c) Assessment of the dynamic range and the sensitivity of the scRNA‐seq protocol. Mean normalized read counts (b) and mean detection rate (c), respectively, across all the single yeast cells that pass the initial quality control (*n* = 125, Table S1) as a function of the (Log_10_) absolute number of 92 ERCC RNA spike‐in molecules. (d) Correlation between our scRNA‐seq data and our bulk RNA‐seq data from cultures grown in similar condition. The expression levels were quantified as TPM (Transcripts Per Kilobase of exon model per Million mapped reads) and log‐transformed with addition of a pseudo count. (e) Number of genes detected per cell across different scRNA‐seq datasets of *S. cerevisiae* (Gasch et al., 2017; Jariani et al., 2020; Nadal‐Ribelles et al., 2019).

To probe more deeply into the mechanisms underlying this early heterogeneity revealed by single‐cell imaging, we further developed and applied scRNA‐seq for transcriptome profiling of yeast aging (Figure 1a; see Section 4). We first conducted a RLS assay by continually performed manual microdissection of single yeast cells on a solid agar plate (Steffen et al., 2009), resulting in an median lifespan of 23.0 (Figure S1d). In the meantime, we manually isolated single aging yeast cells from the plate at three different time points (2, 16, and 36 h after birth). We placed the cells individually and immediately into a single tube prefilled with lysis buffer containing an external RNA control consortium (ERCC) spike‐in for assessing technical noise. Then a refined Smart‐seq2‐based protocol (Picelli et al., 2014) was performed for yeast aging research (see Section 4).

### 2.2 Technical assessment of scRNA‐seq

In total, we collected 136 single yeast cells with precise age for sequencing. The time points of isolation and number of generations at that time were precisely recorded for each cell (Table S1). After filtering out the cells with a low number of genes detected, insufficient read counts and ERCC‐dominated samples, we finally retained scRNA‐seq data of 125 cells composed of 37, 43, and 45 single cells in the 2‐h (young), 16‐h (early age), and 36‐h (late age) age groups, respectively, for further analysis (Figure S2a–c; see Section 4). Our method yielded, on average 2202 genes detected per cell, which accounts for about one third of the coding genes in budding yeast *S. cerevisiae* (Table S1). According to the analysis of ERCC spike‐in molecules, we realized that the dynamic range spanned five orders of magnitude, and the detection rate was more than 90% for transcripts with an absolute copy number above 10 (Figure 1b,c). We compared our scRNA‐seq data to the bulk RNA‐seq data from cultures grown in similar condition, and our scRNA‐seq quantification can reproduce bulk RNA‐seq data with a correlation of 0.75 and *P* value <2.2 × 10^−16^ (Figure 1d).

We also compared our scRNA‐seq with the scRNA‐seq datasets of *S. cerevisiae* growing in different conditions using different methods published recently (Gasch et al., 2017; Jariani et al., 2020; Nadal‐Ribelles et al., 2019). Overall, we found a good genome‐wide correlation between our scRNA‐seq dataset and three existing scRNA‐seq datasets of *S. cerevisiae*, respectively (Figure S2d). Our scRNA‐seq data has similar gene coverage compared with that from Gasch et al. (2017) using Fluidigm C1 system and Nadal‐Ribelles et al. (2019) using UMI strategy (Figure 1e). The dataset from Jariani et al. (2020) was generated using droplet‐based 10× Genomics Chromium system. It has a lower sensitivity compared with our dataset, but with the highest throughput, detecting a median of 1269 gene transcripts per cell from more than 6000 single cells (Figure 1e).

### 2.3 Cell‐to‐cell transcriptional variability during aging in yeast

**FIGURE 2:**
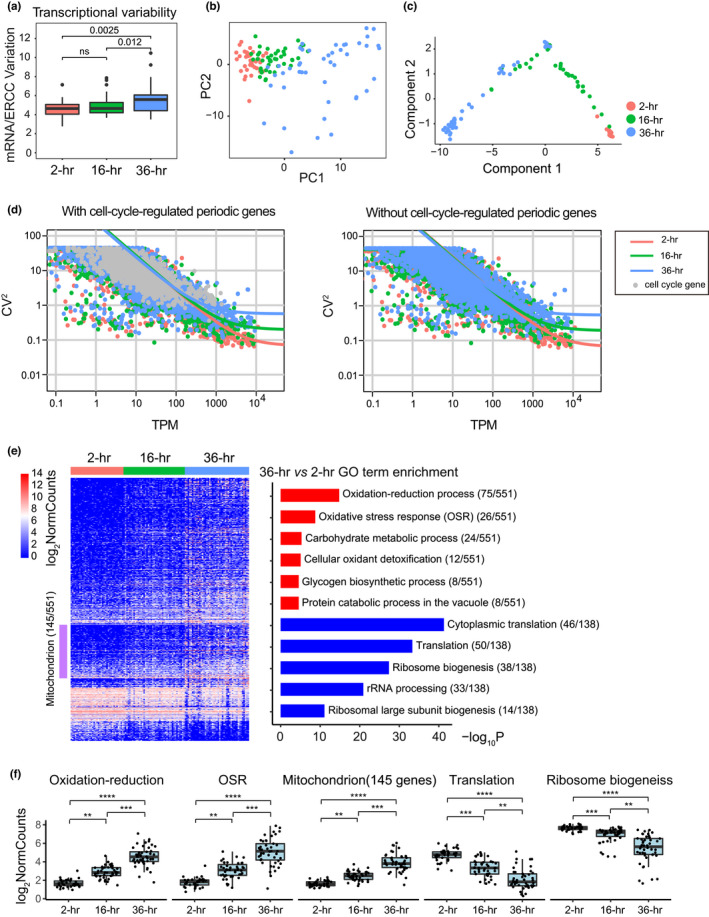
Cell‐to‐cell transcriptional variability and global differential gene expression during aging in yeast. (a) Boxplot showing an increased cell‐to‐cell transcriptional variability during aging in yeast based on a correlation analysis where the transcriptional variability was measured as biological noise over the technical noise (see Section 4). Boxes indicated the first and third quartiles, separated by median line. Whiskers indicated last values within 1.5 × the interquartile range for the box; Wilcoxon *p* values were also shown. (b) PCA plot of single cells (*n* = 125) from different age groups (no cell‐cycle‐regulated periodic genes included as input for PCA). The distribution of single yeast aging cells in the 36‐h late age group was more scattered than that of 2‐h age group and 16‐h early age group, which reflected an increased cell‐to‐cell transcriptional variability. (c) Pseudotime trajectory of single cells (*n* = 125) from different age groups (no cell‐cycle‐regulated periodic genes included as input). The youngest 2‐h age group was very concentrated, whereas the 16‐h early age group and 36‐h late age group were very scattered. (d) Scatter plot of the TPM (Transcripts Per Kilobase of exon model per Million mapped reads) and noise (CV^2^) of genes across different age groups, with (left) or without (right) the cell‐cycle‐regulated periodic genes expression data as input. The solid lines were linear fits of the data and indicated the increase in noise during aging. (e) (left) Heatmap of normalized gene expression of 551 upregulated and 138 downregulated genes in the 36‐h age group compared with 2‐h age group, across different age groups. The purple bar indicated 145 mitochondrial genes that were highly expressed in the 36‐h late age group. (right) Significance of GO terms of biological processes (BP) in upregulated and downregulated genes, respectively, (−log_10_P). (f) Boxplots of the average normalized expression of significantly upregulated and downregulated gene categories identified in **e**, across different age groups. Each black dot in (f) represented a single cell. **p < 5.5 × 10^−7^, ***p < 4.2 × 10^−9^, ****p < 1.6 × 10^−13^, from Wilcoxon rank sum test.

We sought to explore the cell‐to‐cell transcriptional variability within different age groups using scRNA‐seq data. Overall, we observed increased cell‐to‐cell transcriptional variability during aging in yeast based on a correlation analysis in which the transcriptional variability was measured as the biological noise over the technical noise (Enge et al., 2017) (Figure 2a; see Section 4). We verified this increase in cell‐to‐cell transcriptional variability alternatively using a quantitative statistical method (Brennecke et al., 2013) and, respectively, identified 145, 312 and 524 highly variable genes (HVGs) with coefficients of variation (CV) that were significantly higher than those of the ERCC spike‐in reference within each age group (Figure S3a; Table S2; see Section 4). The HVGs were not lowly expressed, therefore it's not likely to be a technical result (Figure S3b; Table S2). Interestingly, by Gene Ontology (GO) analysis of these HVGs using DAVID (Dennis G Jr et al., 2003), the biological processes of cellular iron ion homeostasis and siderophore transport were specifically found to be highly enriched in the 16‐h early age group with high statistical significance, implying an early heterogeneity during aging in yeast with regard to iron transport (Table S2).

Because all of the aging single yeast cells analyzed did not have synchronized cell cycles, we wondered whether and to what extent the cell‐to‐cell transcriptional variability was associated with the cell cycle. We found that 19.3%, 12.8%, and 15.5% of HVGs, respectively, among the 3 age groups were regarded as cell‐cycle‐regulated periodic genes (Granovskaia et al., 2010; Figure S3c). These results are consistent with a recent report of scRNA‐seq in budding yeast that cell‐cycle‐regulated periodic genes were enriched in HVGs (Nadal‐Ribelles et al., 2019). However, the trend of increased cell‐to‐cell transcriptional variability during aging remained even when these cell‐cycle‐regulated periodic HVGs were removed from the 3 age groups (117, 272, and 443 HVGs remained, respectively; Figure S3c). And the remaining HVGs were also highly enriched in the same biological processes such as transport, cellular iron ion homeostasis and siderophore transport (Table S2). We further confirmed this trend by principal component analysis (PCA) and pseudotime analysis. Regardless of whether the cell‐cycle‐regulated periodic genes were included in the scRNA‐seq dataset used as input, the 3 age groups were always successfully separated along the axis of first PCA component and were increasingly dispersed (Figure 2b; Figure S3d); moreover, the top 30 genes based on the absolute loading values for the first PCA component always highly overlapped and were enriched in the biological process of cellular response to oxidative stress, which reflects aging itself rather than the cell cycle (Figure S3e; Table S3); finally, the pseudotime analysis using Monocle (Trapnell et al., 2014) revealed that while the young cells (2‐h) were still very concentrated, the cells of the early age group (16‐h) had already become scattered along the trajectory (Figure 2c; Figure S3f).

The expression noise of a gene in the isogenic cell population is composed of intrinsic and extrinsic factors (Elowitz et al., 2002). A previous study of noise in gene expression coupled to different growth rates also has shown the regimes of expression dominated by either intrinsic factors (low expression) or extrinsic factors (high expression; Keren et al., 2015). To examine the sources of the global transcriptional changes in noise during aging, we plotted the mean and CV^2^ (squared coefficient of variation) of gene expression with linear fits of the data across different age groups. We found that there was a significant increase in noise during aging, either with or without the cell‐cycle‐regulated periodic genes expression included as input (Figure 2d). Interestingly, this increase in noise mainly occurred at higher gene expression (TPM > 100), suggesting that it's contributed by extrinsic factors.

### 2.4 Global differential gene expression during aging in yeast

The scRNA‐seq data also allow us to globally investigate the differential gene expression between age groups. Thus, we conducted a pairwise comparison among the 3 age groups using DESeq2 (Love et al., 2014; Figure S4a; see Section 4). Obviously, more differentially expressed genes were found between the 36‐h late age group and the 2‐h group (Figure S4a, right panel; Table S4). The biological processes of oxidation–reduction and the oxidative stress response (OSR) were highly enriched in the 36‐h group (75 and 26 out of 551 genes, respectively), while translation and ribosome biogenesis were highly enriched in the 2‐h group (50 and 38 out of 138 genes, respectively) based on the GO analysis of biological process using DAVID (Dennis et al., 2003; Figure 2e, right panel). Moreover, 145 out of 551 genes that were highly expressed in the 36‐h late age group compared with the 2‐h group were enriched in mitochondrion as revealed by the GO analysis of cellular components (Figure 2e, left panel; Table S4).

The average normalized gene expression levels across age groups further demonstrated an age‐dependent increase in oxidation–reduction, OSR and mitochondrion as well as a decrease in translation and ribosome biogenesis (Figure 2f). Indeed, these transcriptome changes had already occurred in the 16‐h early age group. Although far fewer differentially expressed genes were found in the 16‐h early age group compared with the 2‐h group (Figure S4a, left panel), early signs of upregulation in oxidation–reduction and downregulation in ribosome biogenesis (15 out of 108 genes and 4 out of 10 genes, respectively) were observed (Figure S4b; Table S4). Notably, the global differentially expressed genes between age groups and their enriched GO categories from our scRNA‐seq data were found to coincide well with a recent report of transcriptome changes during aging in yeast (Hendrickson et al., 2018), and were even partially consistent with another proteome analysis of aging in *C. elegans* (Walther et al., 2015), although they were both based on bulk population analysis. These aging associated GO categories analyzed by DAVID were also confirmed by ClusterProfiler (Yu et al., 2012; Figure S5a‐f).

### 2.5 Differential gene expression between slow‐ and fast‐dividing age subgroups

**FIGURE 3:**
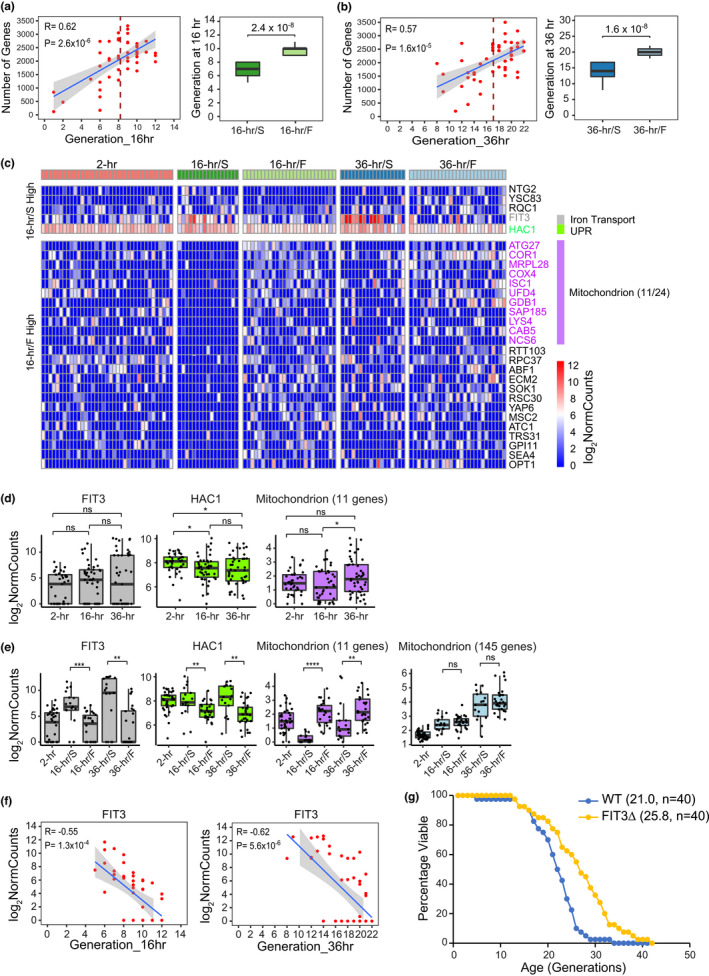
Differential gene expression between slow‐ and fast‐dividing age subgroups. (a) (left) Correlation of the number of genes detected and the generation of single cells in the 16‐h early age group. Each red dot represented a single cell with the number of genes detected and its generation at 16 h. Blue line was a linear fit with gray area indicating 0.95 confidence interval; correlation coefficient (*R*) and *p* value (*p*) were also shown. The dashed line indicated the mean generation. The plot showed a positive correlation between the number of genes detected and the generation at 16 h among individual cells. (right) Boxplot of generation between early age subgroups 16‐h/S and 16‐h/F that were split by the mean generation of 16‐h early age group; Wilcoxon *p* value was shown. (b) (left) Correlation of the number of genes detected and the generation of single cells at 36‐h late age group and (right) Boxplot of generation between late age subgroups 36‐h/S and 36‐h/F that were split by the mean generation of 36‐h late age group, plotted same as in (a). The cells with the number of genes below 1000 plotted in both (a) and (b) were discarded in the rest analysis. (c) Differential gene expression analysis between early age subgroups 16‐h/S and 16‐h/F. The heatmap showed normalized gene expression of statistically significant (Log_2_|FC| > 1 and *p*_adj_ < 0.05) upregulated and downregulated genes in early age subgroup 16‐h/S compared with 16‐h/F, across different age subgroups. (d) Boxplots of normalized expression of significant differentially expressed genes of *FIT3*, *HAC1*, and gene category of mitochondrion identified in (c) across different age groups. (e) Boxplots of normalized expression of significant differentially expressed genes of *FIT3*, *HAC1*, and gene categories of mitochondrion, respectively, identified in (c) and Figure 2f across different age subgroups. Each black dot in (d) and (e) represented a single cell. *p and **p < 0.05, ***p < 0.01, ****p < 6.1 × 10^−5^, and “ns” means not significant, from Wilcoxon rank sum test. (f) Correlation of normalized gene expression of *FIT3* and the generation of single cells in the 16‐h early age group and 36‐h late age group, respectively. Each red dot represented a single cell. Blue line was a linear fit with gray area indicating 0.95 confidence interval; correlation coefficient (*R*) and *p* value (*p*) were also shown. The plot showed a negative correlation for both age groups. (g) Survival curve of WT and *FIT3Δ*. The number in the parenthesis represented the mean RLS and “*n*” indicated the number of cells assayed for RLS of each strain.

The number of genes detected per cell within age groups was found to be positively correlated with the generation, suggesting another facet to understand the heterogeneity of cell divisions during aging in yeast, and the 16‐ and 36‐h age groups were thus split by their respective mean generation into slow‐dividing (16‐h/S, 36‐h/S) and fast‐dividing (16‐h/F, 36‐h/F) age subgroups (Figure 3a,b; Table S1). Comparing the early age subgroups of 16‐h/S and 16‐h/F by DESeq2(Love et al., 2014) with stringent statistical filtering yielded 29 differentially expressed genes, with five highly expressed and 24 weakly expressed in 16‐h/S (Figure 3c; Table S5). *FIT3* and *HAC1* were highly expressed in 16‐h/S. *FIT3*, together with *FIT2* and *FIT1*, as facilitators of iron transport in yeast, encodes a cell wall mannoprotein (Protchenko et al., 2001). These genes were reported to be induced upon iron deprivation or mitochondrial DNA loss (Veatch et al., 2009). *HAC1* is a transcription factor that regulates the unfolded protein response (UPR), and interestingly, one of its regulatory targets is *FIT3* (Cox & Walter, 1996; Hu et al., 2007). Indeed, *FIT3* and *HAC1* were not only highly expressed in 16‐h/S but also in 36‐h/S (Figure 3d,e). Moreover, the gene expression of *FIT3* and *HAC1* negatively correlated with the generation of single cells in the 16‐h age group (*R* = −0.55, −0.38; *p* = 1.3 × 10^−4^, 1.5 × 10^−2^) as well as the 36‐h age group (*R* = −0.62, −0.44; *p* = 5.6 × 10^−6^, 2.2 × 10^−3^; Figure 3f; Figure S6a; Table S5). Gene expression levels of several other iron transporters, including *FIT2* and *FET3* (Protchenko et al., 2001), were also found to be negatively correlated with the generation of single cells in the 16‐ and 36‐h age groups (Figure S6b,c; Table S5). Finally, as single‐gene deletions of *FIT2* and *FET3* were both reported to extend the lifespan in yeast (McCormick et al., 2015), we measured the RLS of yeast after deleting FIT3, and verified that this strain is long‐lived as well (Figure 3g). Collectively, these results reveal a molecular marker of iron transport that can indicate early heterogeneity during aging in yeast and quantitatively predict the lifespan.

Interestingly, we also revealed that 11 out of 24 genes expressed at lower levels in 16‐h/S than in 16‐h/F were enriched in mitochondrion, and these genes were also expressed at lower levels in 36‐h/S than in 36‐h/F (Figure 3c–e; Table S5). This further suggests a relatively poor mitochondrial function in the slow‐dividing cells. Among these 11 weakly expressed mitochondrial genes (Figure 3c), *COR1* is the core subunit of ubiquinol‐cytochrome c reductase which belongs to complexes III and *COX4* is an important component of cytochrome c oxidase which belongs to complexes IV of the mitochondrial inner membrane electron transport chain. It has been reported that mutation of either *COR1* or *COX4* can cause a decrease in respiration, slow cell growth and even shorter lifespan (Allan et al., 2013; Herrmann & Funes, 2005; Marek & Korona, 2013). These 11 mitochondrial genes showed no overlap with the 145 mitochondrial genes that were globally upregulated during aging in yeast (Figures 2e and 3c, Tables S4 and S5); in contrast, no significant differential expression of those 145 mitochondrial genes was observed between the age subgroups (Figure 3e). These results successfully characterize divergent mitochondrial gene expression profiles between age groups and subgroups that would be masked in the bulk population analysis but can be identified by scRNA‐seq.

We performed the correlation analysis between the gene expression and the generation of single cells in the 16‐h early age group. And ribosome biogenesis was found to be enriched (Figure S6d; Table S5). This suggests a downregulation of at least some ribosome biogenesis genes during early aging and it was mainly contributed by the cells from the slow‐dividing age subgroup, which were inclined to be short‐lived (Figure S6e). Meanwhile, genes enriched in translation, mitochondrial translation and glycolytic processes were positively correlated with generation in the 36‐h late age group (Figure S6f). This agrees with the differential gene expression analysis above, suggesting a relatively poor machinery of translation and mitochondrion in the slow‐dividing age subgroups. In summary, these results characterized early and late heterogeneity during aging in yeast at the single‐cell transcriptome level.

### 2.6 Temporal regulation of transcription factor (TF) between age subgroups

**FIGURE 4:**
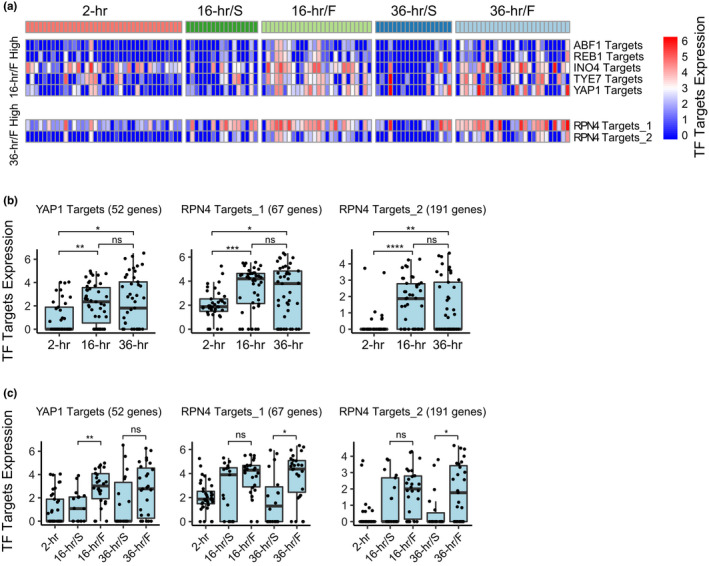
Temporal regulation of transcription factor (TF) between age subgroups. (a) Heatmap showing differential expression of 5 transcription factor targets in the early age subgroup of 16‐h/F compared with 16‐h/S, and 2 transcription factor targets in the late age subgroup of 36‐h/F compared with 36‐h/S, based on first two statistical criteria (see Section 4). (b, c) Boxplots of differential expression of *YAP1* targets that were highly expressed in the early age subgroup of 16‐h/F compared with 16‐h/S, and 2 *RPN4* targets that were highly expressed in the late age subgroup of 36‐h/F compared with 36‐h/S identified by three stringent statistical approaches (see Section 4), across different age groups and subgroups, respectively. Each black dot in (b) and (c) represented a single cell. *p < 0.05, **p < 0.01, ***p < 1 × 10^−3^, ****p < 1 × 10^−4^, and “ns” means not significant, from Wilcoxon rank sum test.

We further investigated the regulatory variation in transcription factors (TFs) between age subgroups, analyzing 634 overlapping TF targets (gene clusters) based on TF binding data of budding yeast (Gasch et al., 2017). To eliminate false‐positives, we performed stringent statistical analysis with three approaches (see Section 4). First, we conventionally compared the median TF targets expression between age subgroups. This led to 16 TF targets that were significantly activated in the 16‐h/F subgroups and 11 TF targets in 36‐h/F subgroups compared with their counterparts, respectively (Figure S7a,b; Table S6). Then, we ran a Wilcoxon rank sum test comparing normalized gene expression levels of each set of TF targets to that of all other detected genes for each cell, taking *P* < 0.0001 as the criterion, followed by intersection with TF targets derived from the conventional analysis. This led to 5 and 2 TF targets that were significantly activated in 16‐ and 36‐h/F, respectively (Figure 4a; Figure S7c; Table S6). Subsequently, we employed correlation analysis between TF target expression and the generation of single cells in the 16‐ and 36‐h age groups, taking *p* < 0.05 as the criterion (Figure S8a,b; Table S6), followed by intersection with TF targets derived from the former two approaches.

Finally, *YAP1* was found to be most significantly active in regulating the early age subgroup of 16‐h/F compared with 16‐h/S (Figure 4b,c), although the other 4 TFs of *ABF1*, *REB1*, *INO4*, and *TYE7* demonstrated a similar trend with less statistical significance (Figure S7d,e). Moreover, two TF targets of *RPN4* were found to be most highly regulated in the late age subgroup of 36‐h/F compared with 36‐h/S (Figure 4b,c). *YAP1* is involved in activating the transcription of antioxidant genes in response to oxidative stress (Temple et al., 2005; Toone & Jones, 1999). The highly activated *YAP1* targets (52 genes; Table S6) in 16‐h/F compared with 16‐h/S suggests that the rapidly dividing single cells, which inclined to be long‐lived, may have a better defense system against oxidative stress than the slow‐dividing cells during early age. *RPN4* is a TF that stimulates proteasome biogenesis for the degradation of damaged proteins (Xie & Varshavsky, 2001). The other two highly activated *RPN4* targets (67 and 191 genes, respectively; Table S6) in the 36‐h/F late age but rapidly dividing subgroup supports the idea that proteasome capacity is critical to maintain the vigor and proteostasis of yeast cells, especially when approaching the end of life, as elevated *RPN4* expression is essential for extending the RLS in yeast (Undine et al., 2011).

To verify the temporal regulatory variation of *YAP1* and *RPN4* between age subgroups, we employed another dataset of TF targets with simultaneous DNA binding and expression evidence (Monteiro et al., 2020), which may further imply TF function. Again, based on this dataset, *YAP1* targets (505 genes; Table S6) were highly expressed in 16‐h/F compared with 16‐h/S early age subgroup and *RPN4* targets (131 genes; Table S6) were highly expressed in 36‐h/F compared with 36‐h/S late age subgroup, both with statistical significance (Figure S9a,b).

Altogether, these findings reveal early and late heterogeneity by distinctive temporal regulation of TFs during aging in yeast and combined with the aforementioned differential gene expression analysis between age groups and subgroups, we successfully depicted a landscape of aging in yeast with unprecedented detail at single‐cell resolution.

## 3 DISCUSSION

Although transcriptome changes during aging in yeast based on bulk population analysis have been reported (Hendrickson et al., 2018; Hu et al., 2014; Leupold et al., 2019; Lindstrom & Gottschling, 2009; Smeal et al., 1996), such analysis at the single‐cell level had not yet been performed. Here, we first identified an early heterogeneity of cell divisions during aging in yeast by single‐cell imaging. Then, we developed and applied scRNA‐seq for single‐cell transcriptome analysis during aging in yeast for the first time.

Using scRNA‐seq technology, we overcame the difficulty of purifying the large number of old cells required for conventional transcriptome analysis during aging in yeast. Our results have unveiled an increased cell‐to‐cell transcriptional variability independent of cell cycle and identified an early heterogeneity during aging in yeast. This also coincides with recent reports of scRNA‐seq in mouse immune cells and human pancreatic cells during aging (Enge et al., 2017; Martinez‐Jimenez et al., 2017). No matter the cell‐cycle‐regulated periodic genes expression data was included as input or not, there was always a significant increase in noise during aging, implying that expect for cell cycle there were some other extrinsic factors contributing to the increase in noise during aging in yeast (Elowitz et al., 2002; Keren et al., 2015; Figure 2d).

By single‐cell transcriptome analysis, we not only successfully recapitulated the results of the bulk population analysis but also teased out specific transcriptional features at the single‐cell resolution that would otherwise be masked in a bulk population. For example, by scRNA‐seq we revealed that while globally there were an age‐dependent upregulation of many mitochondrial genes between age groups, a small number of different but important mitochondrial genes were significantly downregulated in the slow‐dividing age subgroups compared with their fast‐dividing counterparts (Figure 3c–e). This provides novel and unprecedented insights into our understanding of the aging process. We also identified the gene expression of *FIT3* together with several other iron transporter genes, such as *FIT2* and *FET3*, had a negative correlation with the age of single yeast cells from both early and late timepoints. These iron transporter genes are known to be induced upon iron deprivation or mitochondrial DNA loss (Veatch et al., 2009). Moreover, these genes can all extend the RLS in yeast when deleted (McCormick et al., 2015; Figure 3g). These findings are consistent with a report published recently, showing age‐dependent heterogeneity via a *FIT2* reporter that is correlated with vacuolar pH, mitochondrial function, and lifespan in sub‐populations of yeast cells (Chen et al., 2020). Although *HAC1* was highly expressed in the slow‐dividing age subgroups of 16‐ and 36‐h/S compared with their fast‐dividing counterparts (Figure 3e), we did not see the same trend when comparing its targets gene expression between age subgroups (Figure S9c, Table S6). This implies that the highly expressed *HAC1* total RNA from scRNAseq data may not reflect its active form of splicing. In the future, some new methods such as nanopore sequencing with long reads may help address this issue.

Our scRNA‐seq dataset suggests a relatively poor mitochondrial function in the slow‐dividing cells of both early and late age subgroups (Figure 3c,e). This is in accord with the recent work about two aging modes in individual yeast cells: mode 1 with nucleolar decline which inclined to be long‐lived and mode 2 with mitochondrial decline which inclined to be short‐lived (Li et al., 2020). However, presently it remains challenging to disentangle the cause‐effect relationships between mitochondrial dysfunction and early heterogeneity during aging. We keep optimistic that these problems can be solved if the potential of modern single‐cell technologies integrated with other new methods are fully employed.

Based on the scRNA‐seq data and knowledge of TF targets in the budding yeast *Saccharomyces cerevisiae* (Gasch et al., 2017; Monteiro et al., 2020), we also explored TF regulatory variation at the single cell level and found distinctive temporal regulation of TFs during aging in yeast. *YAP1* is a key TF responding to oxidative stress (Temple et al., 2005; Toone & Jones, 1999), and it was highly activated in early age and fast‐dividing subgroup (16‐h/F) compared with its slow‐dividing counterpart (16‐h/S), implicating its vital role during early age, which in turn affects the overall lifespan. *RPN4*, the TF essential for proteasome biogenesis and RLS extension (Undine et al., 2011; Xie & Varshavsky, 2001), was prominently activated in late age and fast‐dividing subgroup (36‐h/F) compared with its slow‐dividing counterpart (36‐h/S), suggesting that the proteasome activity is essential for maintaining the vitality of yeast cells during late age (Figure 4b,c; Figure S9a,b). These aforementioned findings imply that both the mitochondrial dysfunction and the inability to respond to oxidative stress occurred earlier than the decline of proteostasis during aging in yeast, especially in the slow‐dividing age subgroups which inclined to be short‐lived (Figure 3c–e), although the detailed mechanism requires further investigation.

## 4 METHODS

### 4.1 Strains and growth conditions

WT *Saccharomyces cerevisiae* in both BY4741 and BY4742 backgrounds were used for single‐cell imaging analysis. The strain of *Hsp104*‐GFP was derived from the standard GFP strain library in WT BY4741 background. WT BY4742 background was used in scRNA‐seq during aging. WT BY4741 background was used in the replicative lifespan assay of *FIT3*Δ. For single‐cell imaging, the cells were grown in the YPD liquid media before and after loading into the microfluidic chips. For scRNA‐seq during aging and replicative lifespan assay of *FIT3*Δ, the cells were grown on SD solid agar plates.

### 4.2 Single‐cell imaging data analysis

The approach for single‐cell imaging data analysis has been reported in detail elsewhere (Zhang et al., 2012). Yeast cell culture was grown in YPED at 30°C with OD600 of 0.5 before loading into the microfluidic device by a syringe connected to an automatically controlled peristaltic pump. The microfluidic device was mounted on a Nikon TE2000 time‐lapsed microscope by a customized holder. Bright field images were taken once every 10 min throughout the whole life, and fluorescent images were taken once every 2 or 4 h for measuring the *HSP104*‐GFP level. The images were processed by ImageJ and MATLAB.

### 4.3 Dissection and isolation of single cells for RNA‐seq

We first inoculated WT yeast cells onto a solid agar plate with SD media for overnight and followed a standard protocol of replicative lifespan assay by continual (no storage in the 4°C fridge overnight) manual microdissection (Steffen et al., 2009). In detail, we selected relatively young and small sized cells from the yeast colonies and aligned them on the same agar plate. After one and a half hours, we dissected and discarded mother cells, retaining daughter cells as our initial age 0 cells. Then at 3 time points (2, 16 and 36 h after birth), single yeast aging cells from the plate were manually dissected and placed individually into a single tube prefilled with ~4 μl lysis buffer, containing 0.5%Triton, 2.5 μM oligo‐dT, 2.5 mM dNTP (Invitrogen, 1959189), 8000 molecules of external RNA control consortium (ERCC) spike in, 3 × 10^−2^ U/μl zymolyase (ZYMO, E1004‐A), 1 U/μl Recombinant RNase Inhibitor (TaKaRa, AI41189A). Zymolyase was added for efficiently digesting the cell wall and external RNA control consortium (ERCC) spike‐in for assessing technical noise. Then we immediately put lysis tube containing single yeast cell into liquid nitrogen and then stored in a −80°C freezer before the next steps for library preparation. Once we finished the sampling step, we should start the library preparation as soon as possible.

### 4.4 Library preparation for scRNA‐seq

After collecting all the single yeast aging cells, we performed scRNA‐seq based on Smart‐seq2 (Picelli et al., 2014) with fine optimization. To efficiently lyse the single yeast aging cell and avoid possible mRNA degradation, we vigorously vortexed the lysis tubes (~4 μl) for 1 min and spin down in a cold room (4°C). Then we kept the lysis tubes at 30°C for 10 min, followed by 3 min at 72°C. Subsequently, we added the RT reaction mix (RT‐buffer and Invitrogen SuperScript II) for reverse transcription. Reverse transcription was carried out at 42°C for 90 min first, followed by 12 rounds of temperature cycling between 50 and 42°C with 2 min each. The reaction was heat inactivated at 70°C for 15 min and then cooled down to 4°C. The oligo‐dT and TSO primers used here were biotinylated to avoid potential production of excessive primer dimers and concatamers. After RT, the cDNA were amplified between 20 and 25 cycles using KAPA HiFi enzyme. After cDNA amplification, the samples were purified using Agencourt AMPure XP beads at 0.8× bead concentration and quantified using Qubit Hs Assay (Life Technologies). We also checked the samples by a fragment analyzer to confirm the clean peak at ~1 kb before subsequent processing. 1–2 ng of cDNA was subjected to a tagmentation‐based protocol (Vazyme TruePrep Kit) with 10 min at 55°C and dual index amplification for the library with 8–12 cycles. The final libraries were purified twice using AMPure XP beads at 0.8× bead concentration and resuspended in 15–20 μl elution buffer. Libraries were then quantified using Qubit Hs Assay before pooling for sequencing. Sequencing was performed in paired‐end mode using Illumina NextSeq.

### 4.5 scRNA‐seq data pre‐processing and filtering

Paired‐end reads were mapped to the S288c *Saccharomyces cerevisiae* genome R64 version (www.yeastgenome.org) with ERCC spike‐in sequences added using HISAT2 (version 2.1.0). Resulting bam files were sorted and indexed using samtools (version 1.1). Final read counts mapped to genes were extracted using FeatureCounts. Sequenced single yeast aging cells were removed from the analysis if they have <1000 genes detected and 40,000 total mapped reads per cell, or if the proportion of ERCC spike‐ins to total‐mapped reads was >0.74. After filtering, a scRNA‐seq data set with 125 single yeast aging cells was used for the subsequent analysis.

### 4.6 Normalization

Unless noted, normalization of raw read counts was done using the DESeq2 (Love et al., 2014) package (v.1.22.2) in R. The size factor was computed by a formula embedded in DESeq2 for each cell based on the raw read counts matrix of all samples. Then these size factors were applied for normalizing different cells and finally the gene expression values are presented in the log_2_ space (log_2_NormCounts).

### 4.7 Estimation of cell‐to‐cell transcriptional variability and identification of highly variable genes

We used two methods to estimate the cell‐to‐cell transcriptional variability during aging in yeast. The first was a correlation‐based method modified from Enge et al. (2017), where the transcriptional noise was expressed as biological variation over technical variation. First, we calculated the biological variation *b*_*ij*_ = 1‐cor(*x*_*ij*_, *u*_*i*_), where *u*_*i*_ was the mean gene expression vector for the single cells in age group of i (2, 16, and 36 h), and *x*_*ij*_ was the gene expression vector of cell *j* in the age group of *i*. Next, we calculated the corresponding technical variation *t*_*ij*_ = 1−cor(${x_{\mathrm{ij}}}^{\text{contr}}$, *u* ^contr^) where ${x_{\mathrm{ij}}}^{\text{contr}}$ and *u* ^contr^ are the expression vector and mean expression vector of the ERCC spike‐in controls. Finally the measurement of *b*_*ij*_/*t*_*ij*_ which reflected the biological noise as a fraction of technical noise for each cell was used for boxplot across different age groups as shown in Figure 1b. The second method was based on quantitative statistics reported previously (Nadal‐Ribelles et al., 2019; see Supplementary Note 6 of Brennecke et al. (2013) for details of the statistical model). Briefly, to infer the genes that were highly variable within each age group, a linear regression model was applied to fit the relationship between the squared coefficient of variation (CV^2^) and the mean expression of ERCC spike‐ins, and only genes with biological squared coefficient of variation >0.25 (CV^2^ > 0.25) and FDR < 0.1 after multiple testing correction were regarded as HVGs.

### 4.8 Differential gene expression and GO analysis

The differential gene expression analysis between pairwise age groups and subgroups was based on DESeq2 (Love et al., 2014) with default parameters, taking log_2_FC > 1 and adjusted *p* value <0.05 as significant. GO analysis of these differentially expressed genes was performed by functional annotation tool of DAVID (Dennis G Jr et al., 2003) that classify the ontology of each gene into biological process or cellular component. The GO term enrichment results derived from DAVID were further verified alternatively by the R package of ClusterProfiler (Yu et al., 2012).

### 4.9 Statistical analysis of regulation of transcription factor between age subgroups

To identify transcription factors with distinct regulation between age subgroups, three statistical approaches were applied stringently. The first one was to conventionally compare the median TF targets expression between age subgroups. We took log_2_FC (FoldChange) of the median TF targets expression between age subgroups >1 (log_2_FC > 1) and a welch *t* test *p* value <0.01 as significant, which resulted in 16 and 11 TF targets, respectively, that were significantly activated in the age subgroups of 16‐ and 36‐h/F compared with to their counterparts (Figure S7a,b; Table S6). The second one was to further run a Wilcoxon rank sum test for each single cell that compare internally the normalized gene expression levels of each set of TF targets to all other detected genes for that cell, taking *p* < 0.0001 as criterion (indicated as regulon activity “on”), followed by intersection with TF targets derived from the first approach. This approach was similar with that from Gasch et al. (2017). The last one was to correlate the TF targets expression with the generation of single cells in the age groups of 16‐ and 36‐h, respectively, taking *p* < 0.05 as criterion, followed by intersection with TF targets derived from the former two approaches to avoid potential false‐positive results.

### 4.10 PCA analysis

Raw read counts matrix with or without cell‐cycle‐regulated periodic genes (Granovskaia et al., 2010) were used as inputs for PCA by Seurat (Butler et al., 2018). When the cell‐cycle‐regulated periodic genes were included, Seurat generates 631 common variable genes of all 125 single yeast aging cells, whose normalized read counts are further applied for PCA. When the cell‐cycle‐regulated periodic genes were excluded, Seurat generated 599 common variable genes of all 125 single yeast aging cells for PCA.

## AUTHOR CONTRIBUTIONS

Y.Z. and Y.H. conceived and designed the project. Y.Z., J.W., Y.S., S.J., X.Z., and G.K.A. conducted the experiments. Y.Z., J.W., B.K., Q.L., J.W., X.Z. and Y.H. analyzed the data. Y.Z., J.W., B.K., X.Z., and Y.H. wrote the manuscript with the help from all other authors.

## CONFLICT OF INTEREST

The authors declare no conflict of interest.

## Supporting information


Figure S1

Table S1

Table S2

Table S3

Table S4

Table S5

Table S6

Video S1

## Data Availability

scRNA‐seq data generated in this study has been uploaded to Gene Expression Omnibus under accession number GSE210032.
