# Supplementary material for: Single‐cell RNA‐seq reveals early heterogeneity during aging in yeast
Source: Aging Cell. 2022 Oct 1;21(11):e13712. doi: 10.1111/acel.13712 (PMC9649600; doi:10.1111/acel.13712)

Figure S1, Zhang et al.

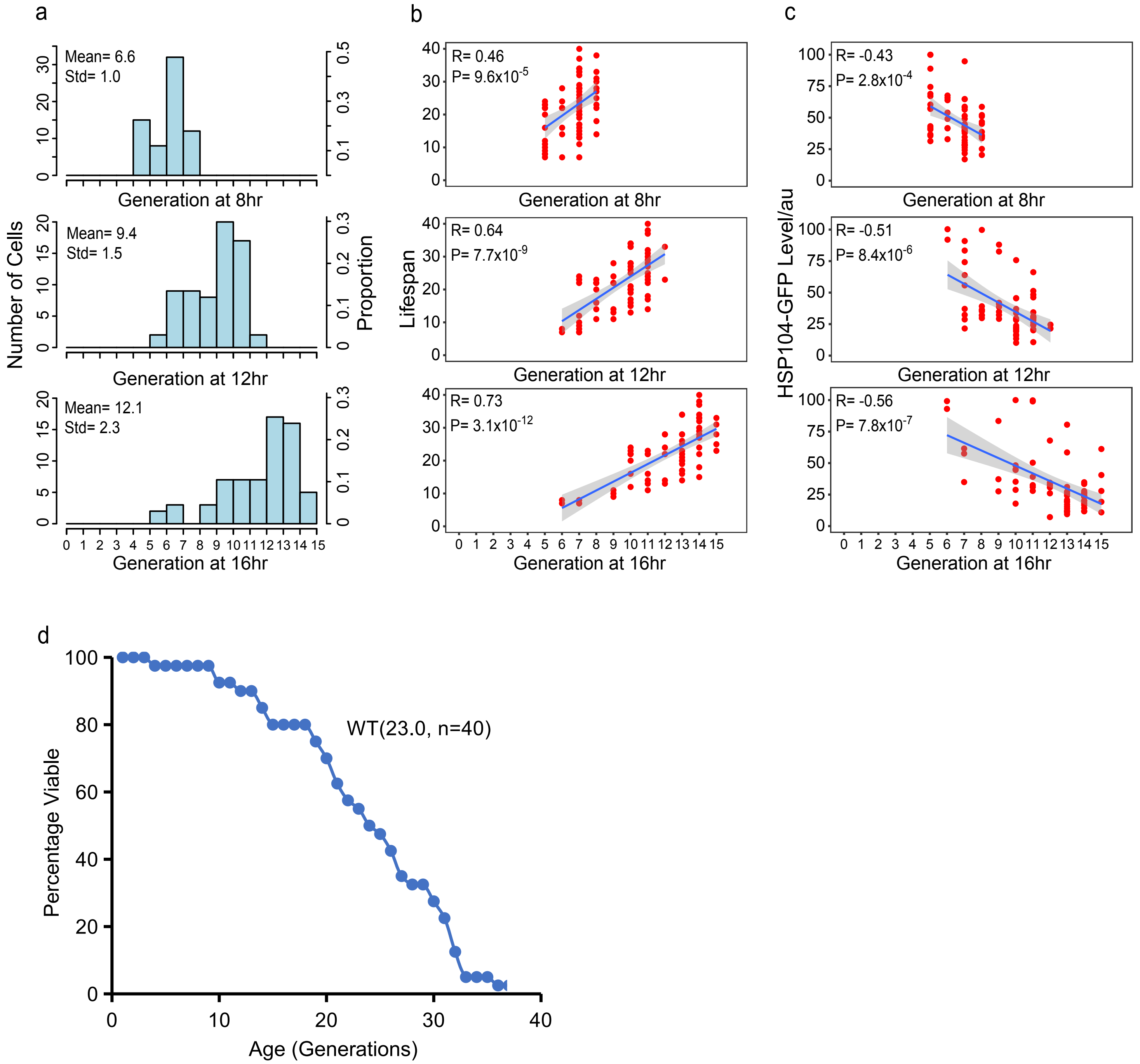

Figure S2, Zhang et al.

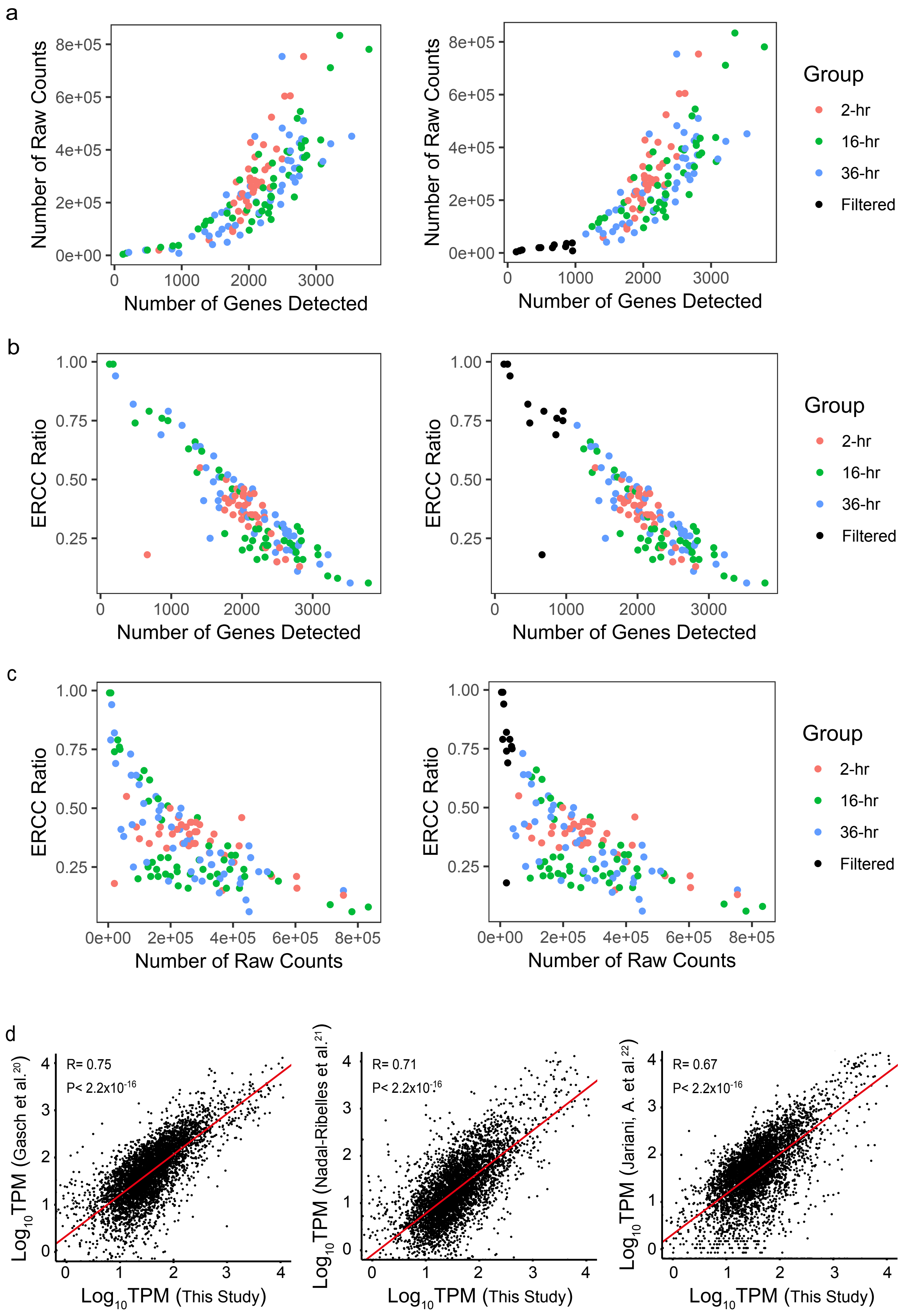

Figure S3, Zhang et al.

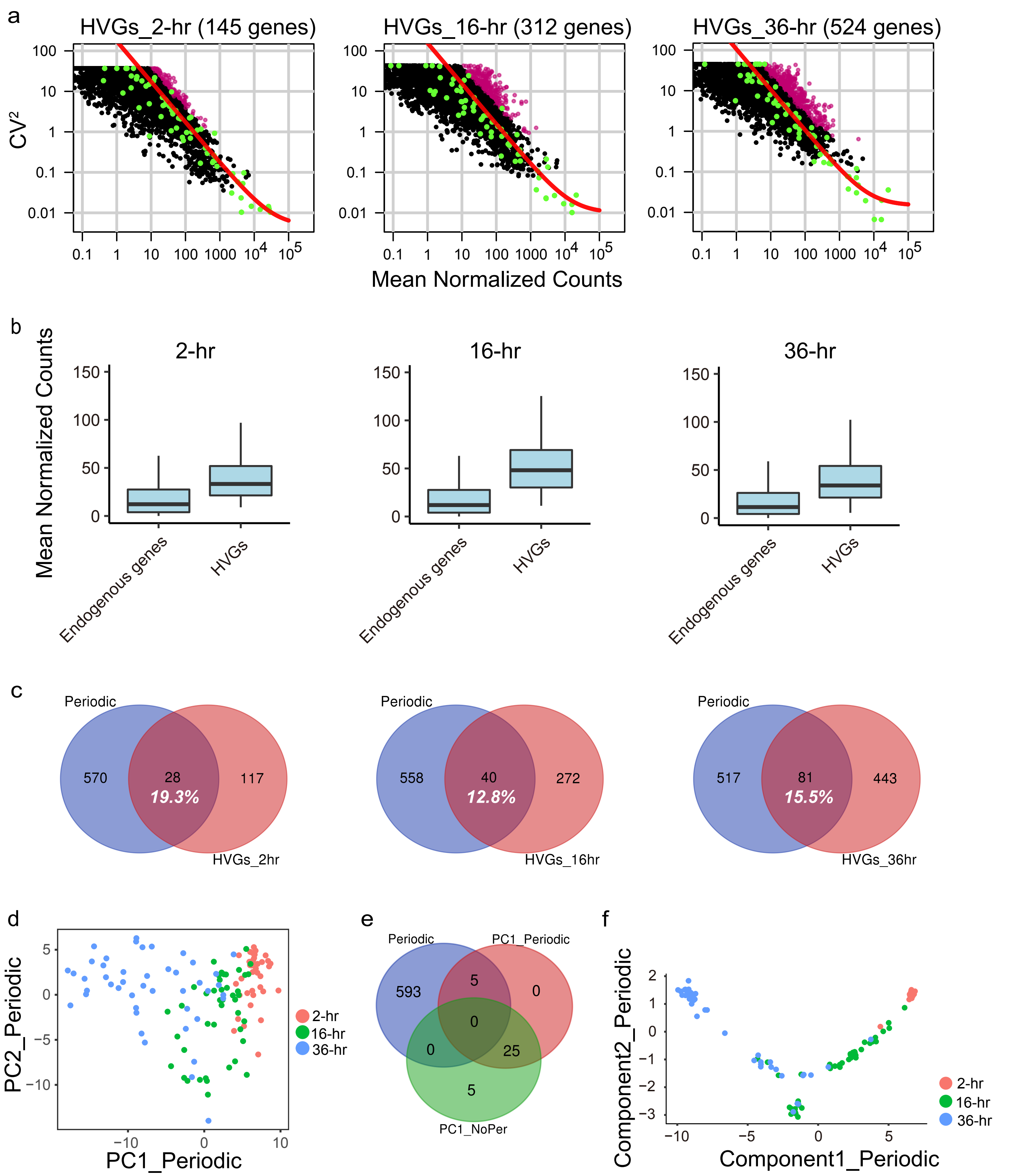

Figure S4, Zhang et al.

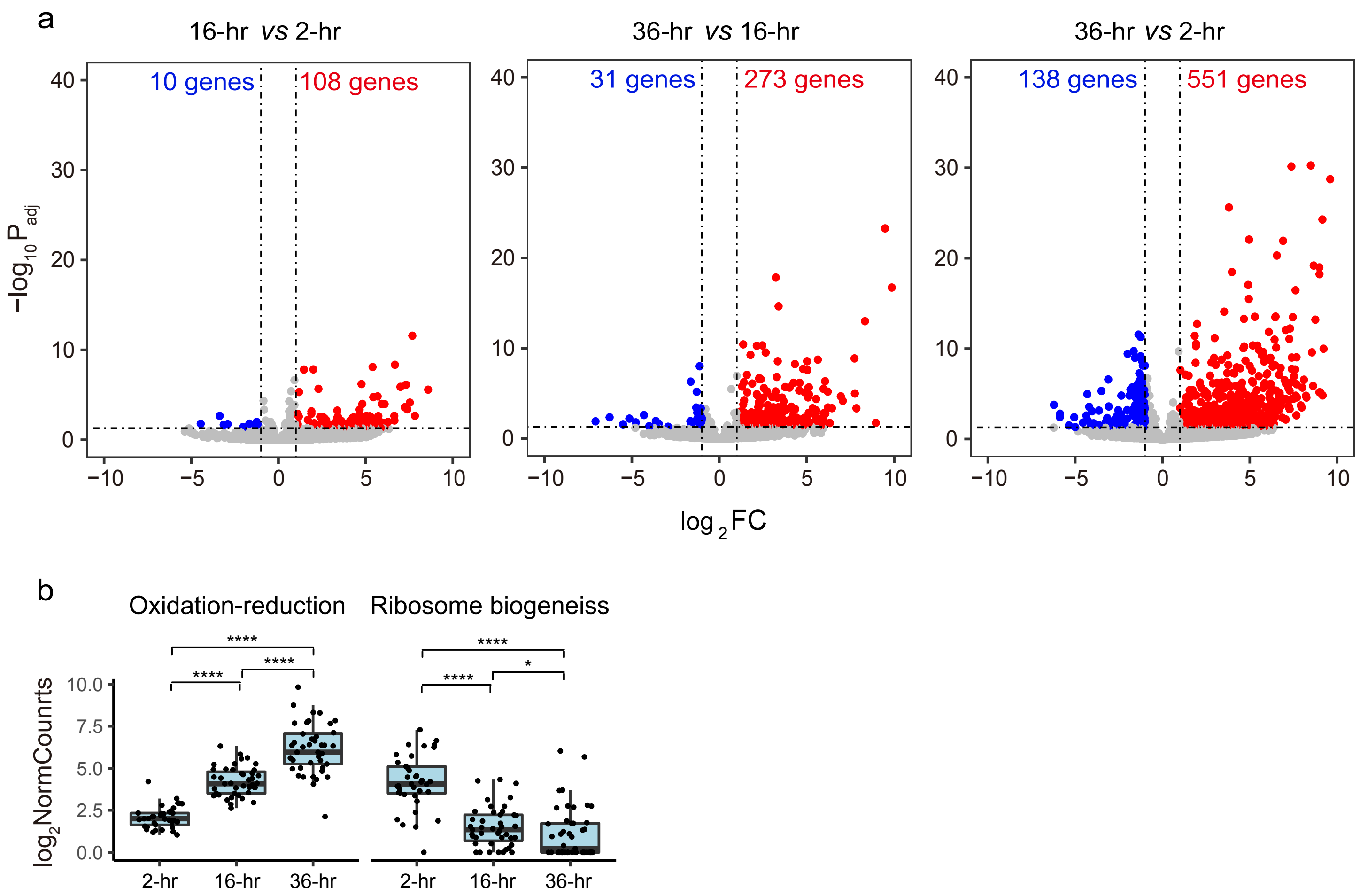

Figure S5, Zhang et al.

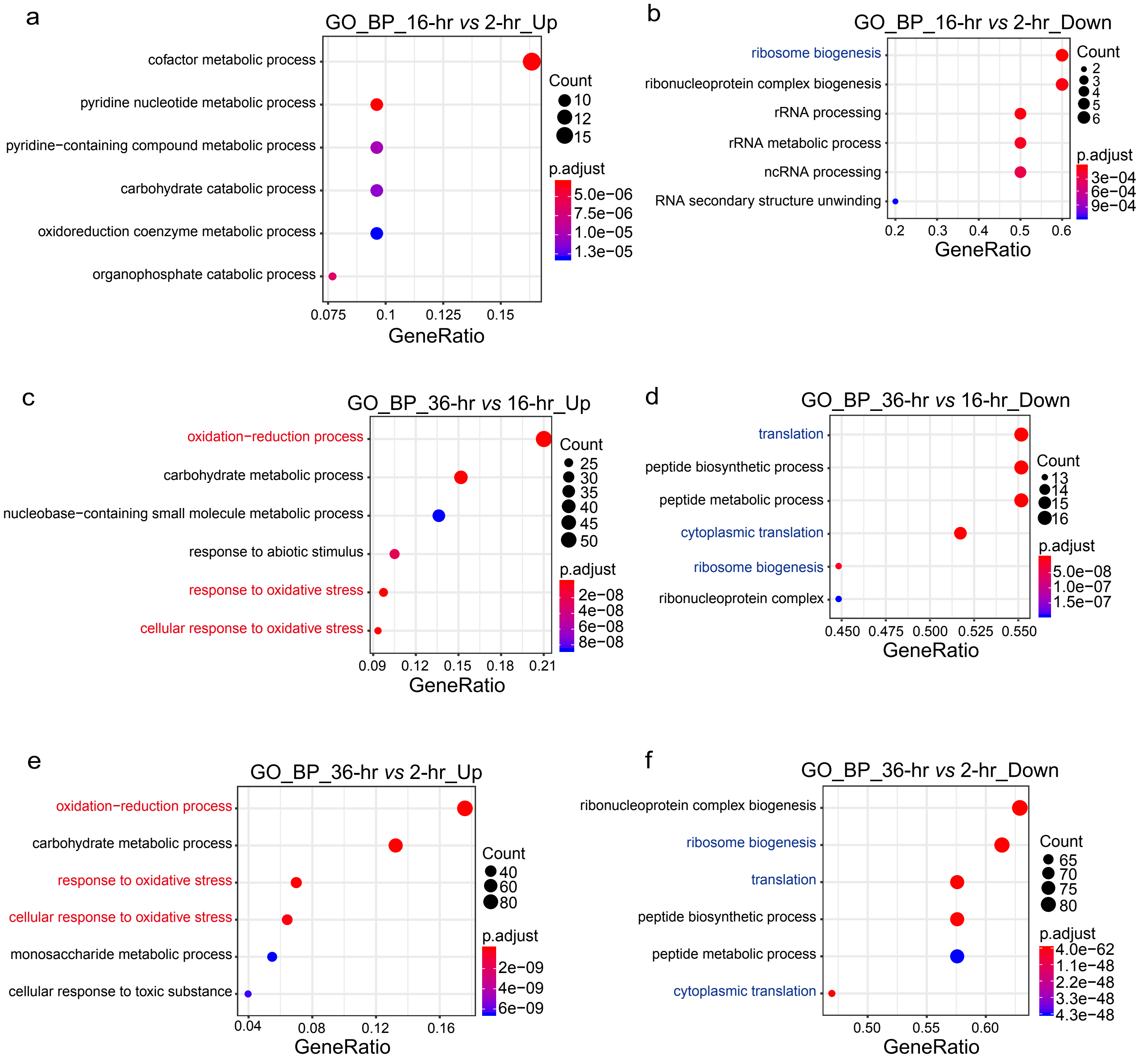

Figure S6, Zhang et al.

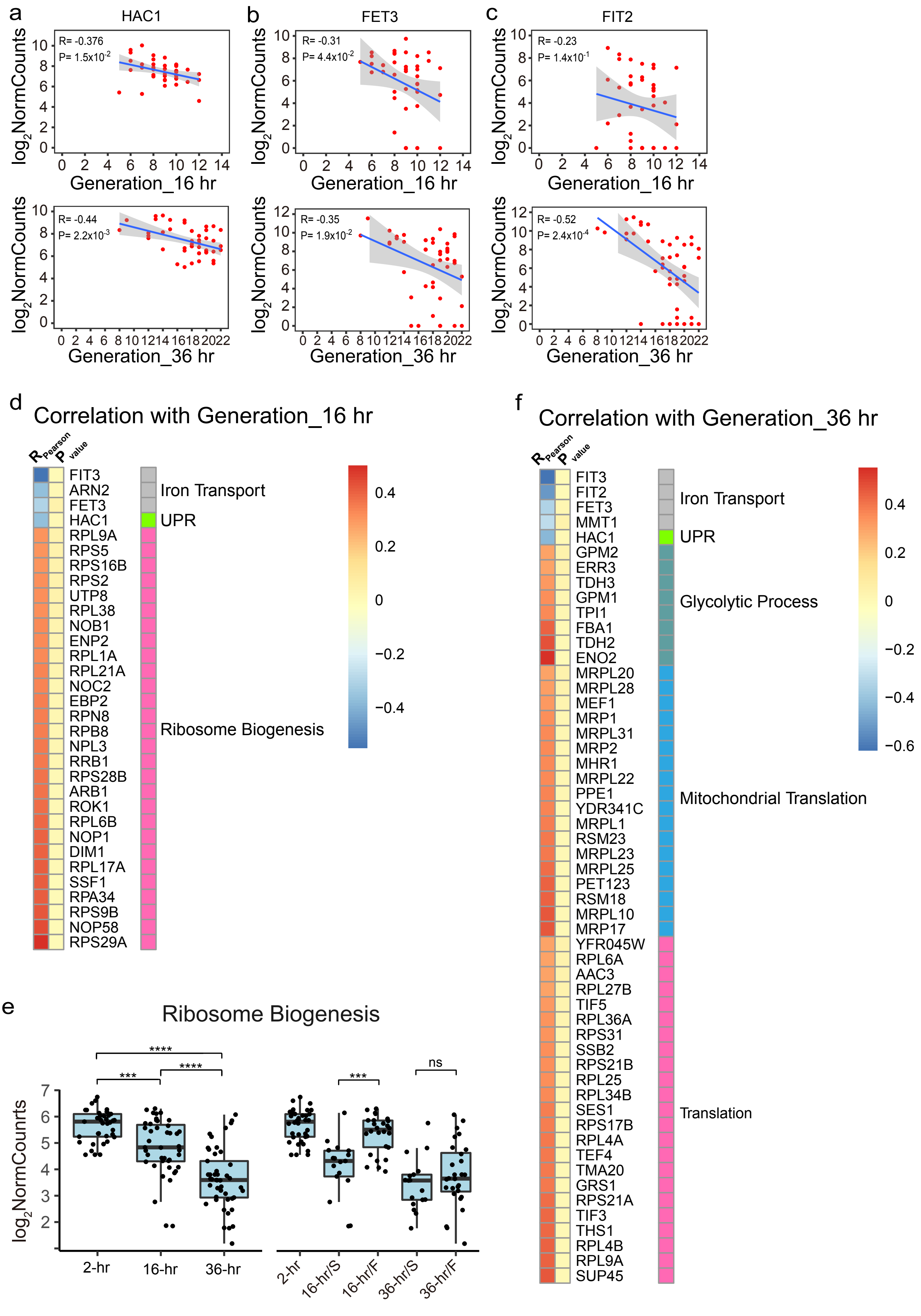

Figure S7, Zhang et al.

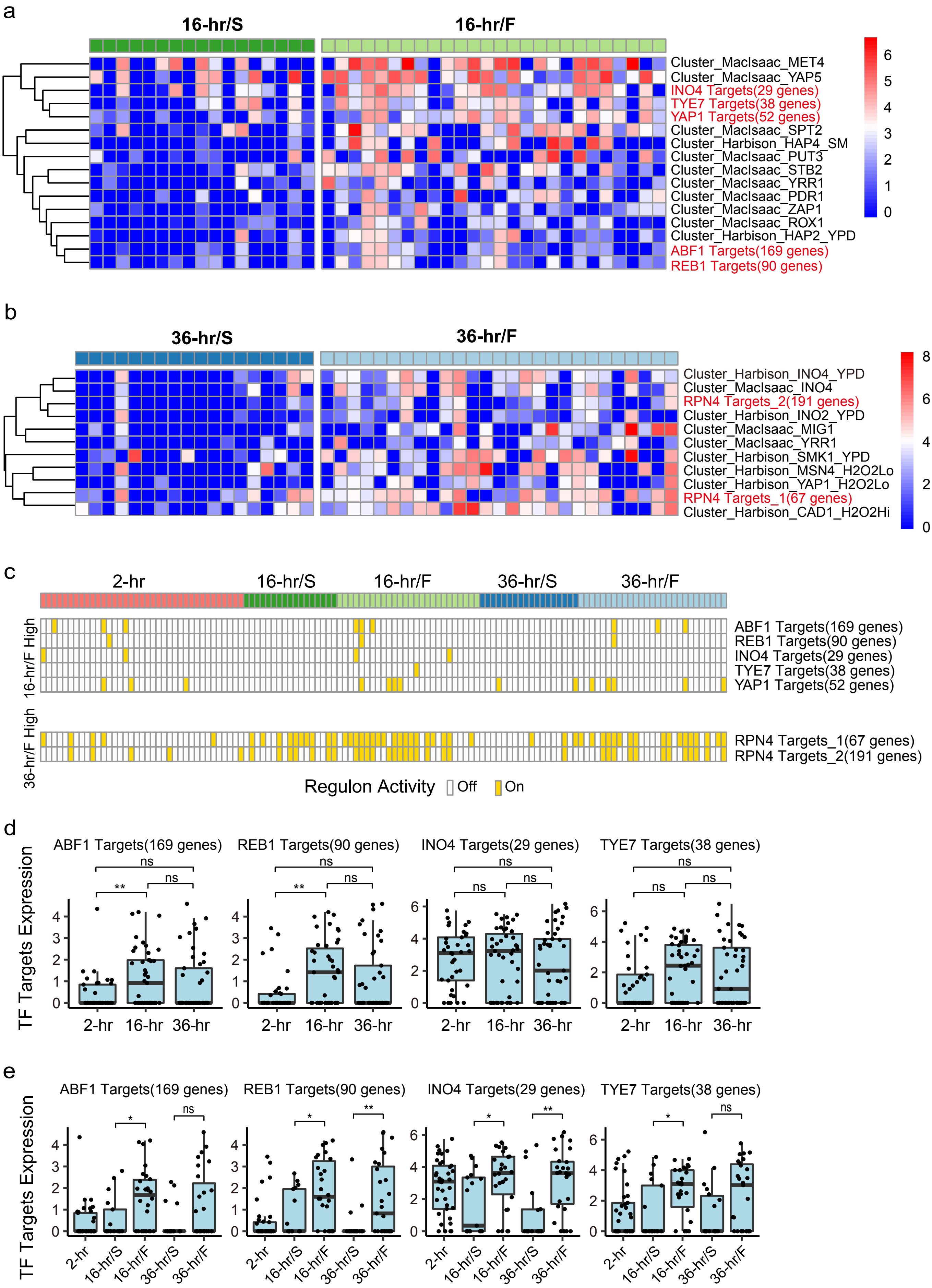

Figure S8, Zhang et al.

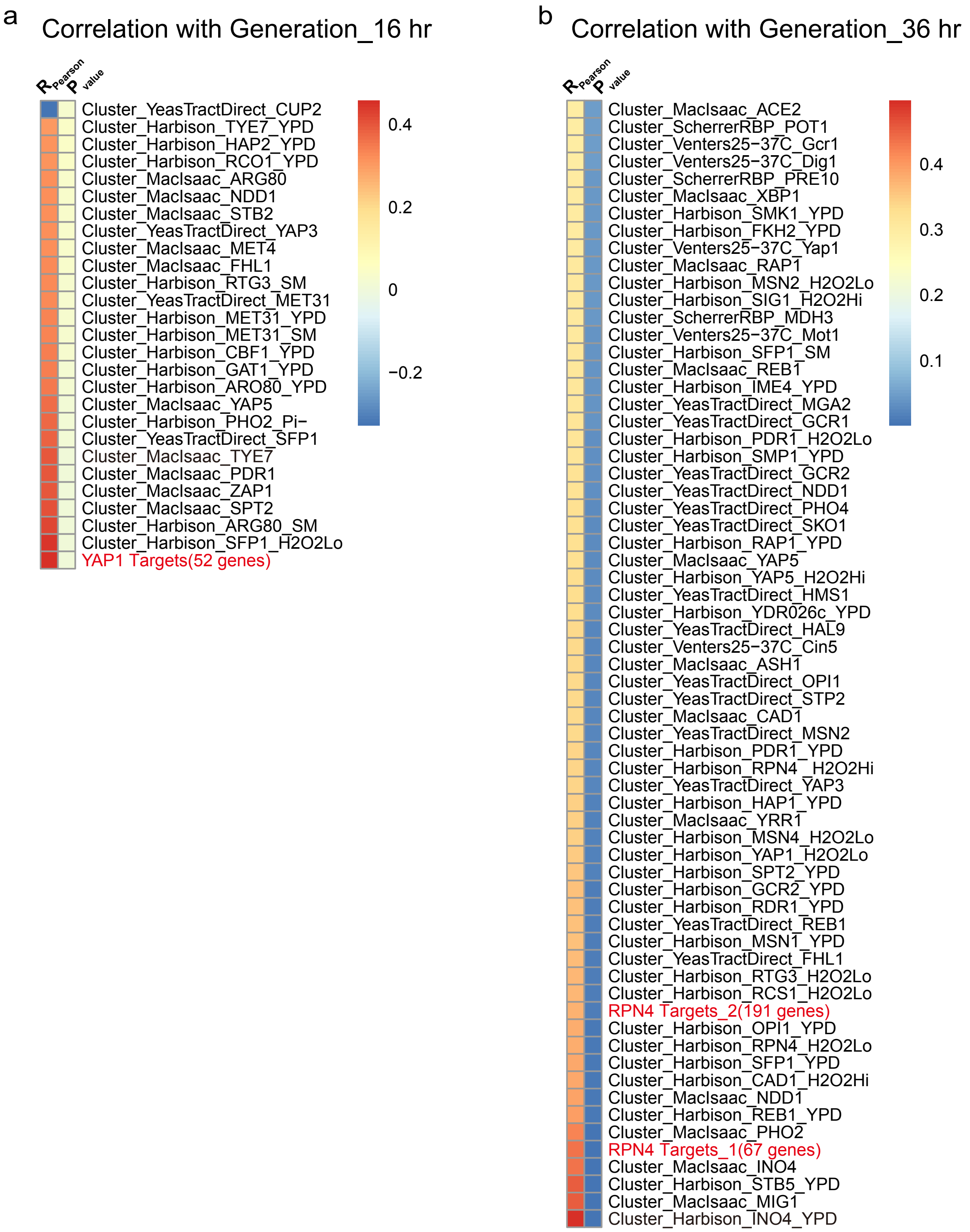

Figure S9, Zhang et al.

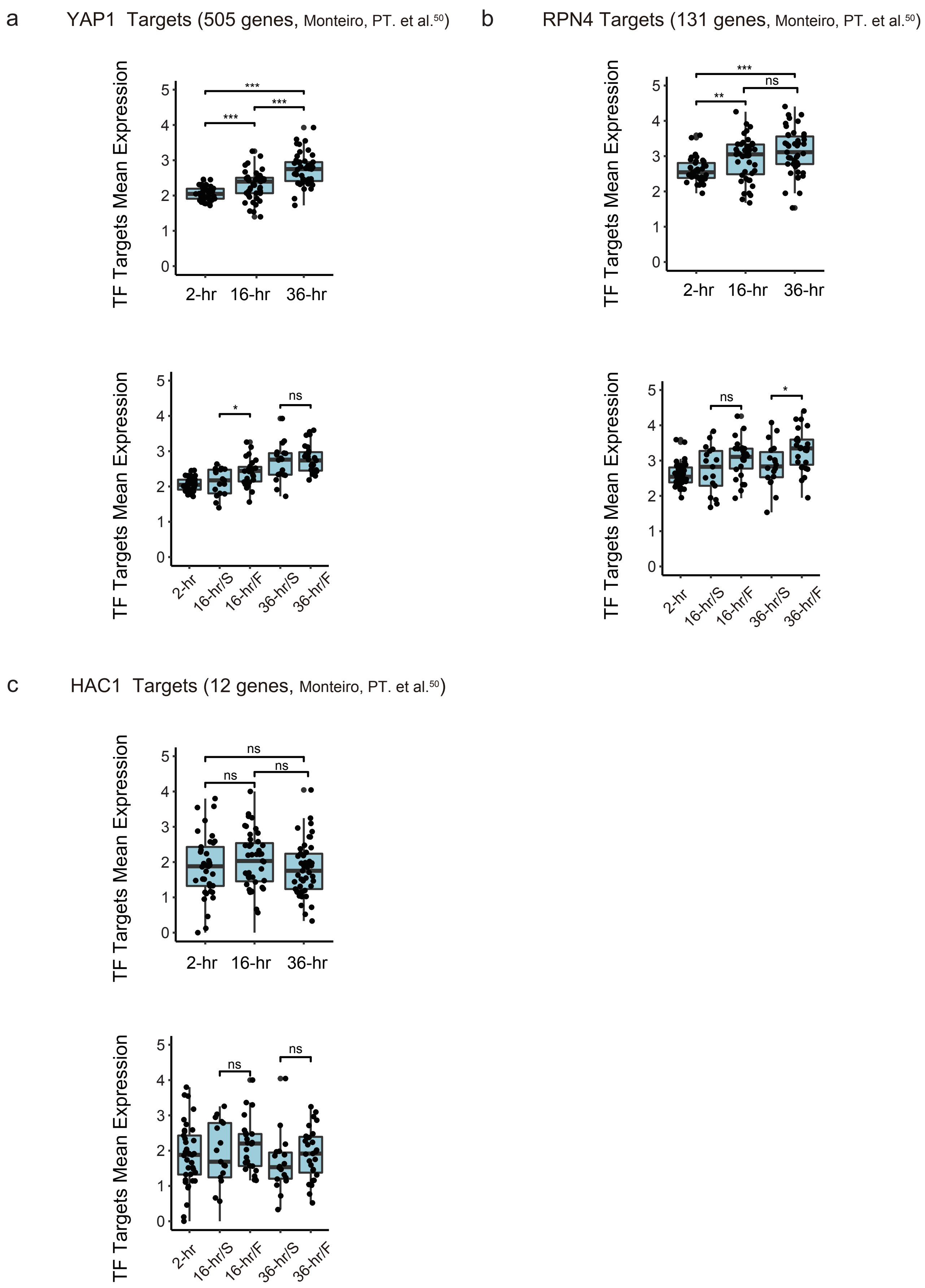

Supplement: Supplementary file 1 — Figure S1 [file ACEL-21-e13712-s004.pdf]
